# Staurosporine augments EGF-mediated EMT in PMC42-LA cells through actin depolymerisation, focal contact size reduction and Snail1 induction – A model for cross-modulation

**DOI:** 10.1186/1471-2407-9-235

**Published:** 2009-07-15

**Authors:** Honor J Hugo, Razan Wafai, Tony Blick, Erik W Thompson, Donald F Newgreen

**Affiliations:** 1 Embryology Laboratory, Murdoch Children's Research Institute, Royal Children's Hospital, Parkville, Australia; 2 VBCRC Invasion and Metastasis Unit, St. Vincent's Institute of Medical Research, Melbourne, Australia; 3 University of Melbourne Department of Surgery, St. Vincent's Hospital, Melbourne, Australia

## Abstract

**Background:**

A feature of epithelial to mesenchymal transition (EMT) relevant to tumour dissemination is the reorganization of actin cytoskeleton/focal contacts, influencing cellular ECM adherence and motility. This is coupled with the transcriptional repression of E-cadherin, often mediated by Snail1, Snail2 and Zeb1/δEF1. These genes, overexpressed in breast carcinomas, are known targets of growth factor-initiated pathways, however it is less clear how alterations in ECM attachment cross-modulate to regulate these pathways. EGF induces EMT in the breast cancer cell line PMC42-LA and the kinase inhibitor staurosporine (ST) induces EMT in embryonic neural epithelial cells, with F-actin de-bundling and disruption of cell-cell adhesion, via inhibition of aPKC.

**Methods:**

PMC42-LA cells were treated for 72 h with 10 ng/ml EGF, 40 nM ST, or both, and assessed for expression of E-cadherin repressor genes (Snail1, Snail2, Zeb1/δEF1) and EMT-related genes by QRT-PCR, multiplex tandem PCR (MT-PCR) and immunofluorescence +/- cycloheximide. Actin and focal contacts (paxillin) were visualized by confocal microscopy. A public database of human breast cancers was assessed for expression of Snail1 and Snail2 in relation to outcome.

**Results:**

When PMC42-LA were treated with EGF, Snail2 was the principal E-cadherin repressor induced. With ST or ST+EGF this shifted to Snail1, with more extreme EMT and Zeb1/δEF1 induction seen with ST+EGF. ST reduced stress fibres and focal contact size rapidly and independently of gene transcription. Gene expression analysis by MT-PCR indicated that ST repressed many genes which were induced by EGF (EGFR, CAV1, CTGF, CYR61, CD44, S100A4) and induced genes which alter the actin cytoskeleton (NLF1, NLF2, EPHB4). Examination of the public database of breast cancers revealed tumours exhibiting higher Snail1 expression have an increased risk of disease-recurrence. This was not seen for Snail2, and Zeb1/δEF1 showed a reverse correlation with lower expression values being predictive of increased risk.

**Conclusion:**

ST in combination with EGF directed a greater EMT via actin depolymerisation and focal contact size reduction, resulting in a loosening of cell-ECM attachment along with Snail1-Zeb1/δEF1 induction. This appeared fundamentally different to the EGF-induced EMT, highlighting the multiple pathways which can regulate EMT. Our findings add support for a functional role for Snail1 in invasive breast cancer.

## Background

Breast cancer is the most common malignancy in women, accounting for 18% of all cancers, and is the leading cause of cancer deaths in women worldwide. Most of these deaths are due to metastatic disease [1]. An increasingly accepted concept is that epithelial to mesenchymal transition (EMT), which is a normal developmental program, is an important preliminary step in metastasis [2,3].

EMT involves a range of cellular phenotypic and functional changes: cell-cell adhesion is disrupted and a more dynamic cell-matrix adhesion is enhanced, polarity and cytoskeletal organization change, ECM molecule synthesis and assembly are altered and an up-regulation of the synthesis and activity of extracellular proteases occurs [4-9].

An early central event in many EMTs is the downregulation of the homophilic cell-cell adhesion molecule E-cadherin. This can be mediated by Snail family proteins which repress E-cadherin transcription by binding the E-box E-pal element in the E-cadherin promoter, a mechanism of action shared by other E-cadherin repressors implicated in carcinogenesis such as Zeb1/δEF1, Zeb2/SIP1 and Twist [10-12]. Indeed, these E-cadherin repressors may not be independent since transfection of Snail1 into E-cadherin positive cells induces a full EMT including the induction of Zeb1/δEF1 [10,13-15].

The Snail1 and Snail2 genes are highly homologous, and in certain circumstances can replace each other functionally. For example, the consequences of Snail2 knockdown in avian embryonic neural crest can be avoided by transfection of Snail1 [16]. However, they also have somewhat distinct roles. Snail1 is essential for mouse gastrulation [17,18], whereas Snail2 knockout mice are viable [19].

In embryonic EMTs, induction by growth factors is a common theme. For example, neural crest EMT follows exposure of neural epithelia to FGFs, Wnts and BMP4 [20]. Several growth factors involved in EMT (EGF, HGF, IGF, FGF and TNFα) activate PI3K and Ras via signaling through their corresponding receptor tyrosine kinases, which in turn activate MAPK leading directly to Snail1/2 upregulation [8,21]. Epidermal growth factor (EGF) is a potent stimulator of EMT in several cell types, and the EGFR has been shown to directly interact with β-catenin, leading to the tyrosine phosphorylation of β-catenin and disruption of cadherin-dependent junctions [22-24]. Endocytosis of E-cadherin results in the release of β-catenin to act on the Wnt pathway, resulting in *Snail *gene transcription and further E-cadherin repression [25]. On the other hand, engaged E-cadherin complexes in the intact adherens junction directly inhibit the activity of the EGFR by inhibiting transphosphorylation of Tyr845 [26]. EGF induces EMT-like changes in the PMC42-LA breast carcinoma cell line, shown by gene expression changes and acquisition of motility [27], are reviewed in [28].

This is an example of the most direct scheme for EMT coordination which involves growth factor signaling to initiate expression of EMT transcription factors which then control EMT motor molecules [21,29]. However, EMT is not likely to be simply a top-down process because there is evidence from embryonic systems that modulating cellular motor molecules such as actin (cytoskeleton), cadherins (cell-cell adhesion) or integrins (adhesion to the ECM) can individually create cellular instability causing or predisposing to EMT-like changes. Moreover, these apparent EMTs may be so rapid as to preclude initiation by gene expression [30-32]. We proposed that the alteration in an embryonic epithelium or carcinoma cells of only a few of the components of the EMT response, for example the physical alteration of cell-cell, cell-ECM or actin cytoskeleton by environmental signals could, by cross-modulation, kindle more EMT elements [32]. This could account for the coordination of the complex molecular changes seen in EMT. Moreover, since irreversible genomic changes are not involved in most steps, it could also fit with the otherwise inexplicable redifferentiations to form epithelia seen in some secondary metastases from invasive tumours [33,34].

Staurosporine (ST) is a serine/threonine kinase inhibitor that induces extremely rapid, coordinated and sustained EMT in embryonic neural epithelial cells [31]. The mechanism is likely to be inhibition of atypical PKC (aPKC) which is part of the Par-3/6-cdc42 complex at the nexus between the cytoskeleton and elements of the cadherin cell-cell adhesion machinery [31,35]. The result is rapid dissociation of F-actin bundles followed by cell-cell adhesion instability. It was concluded that there was a cross-modulation between various EMT effectors [32]. How EMT-like effects via cytoskeletal machinery such as actin may alter signaling pathways when combined with classical EMT inducers (growth factors such as EGF or the TGFβ family) is unknown, and this information would provide important clues as to how cross-modulation may occur.

We have previously shown the epithelial-like human breast carcinoma PMC42-LA subline undergoes an EMT-like change in response to EGF treatment [27], and in response to factors secreted by human breast carcinoma-derived stromal cells [28,36,37]. It also undergoes EMT at the monolayer wound edge in "scratch" assays which is synergistic with EGF-induced EMT (M. Leigh Ackland, unpublished observation). We sought to examine how a combination of EMT inducers (EGF, ST), which each have different cellular effects and speed of action, influence the expression of the E-cadherin repressor gene set and downstream EMT effectors in this breast carcinoma cell line. Finally, we investigated an extensive public database of human primary breast tumours for evidence of clinical implications due to increased expression levels of E-cadherin repressors.

## Methods

### Cell culture

The cell line PMC42-LA, with epithelial characteristics was derived from the mesenchyme-like cell line PMC42 (PMC42-ET) [27], originally derived from a pleural effusion of a patient with metastatic breast carcinoma [38]. PMC42-LA were maintained at 37°C, 5% CO_2 _in RPMI 1640 medium (Thermo Fisher Scientific, Waltham, MA) containing 18 mM HEPES (4- [2-hydroxyethyl]-1-piperazineethanesulfonic acid) and 10% FCS (fetal calf serum, Thermo Fisher Scientific, Waltham, MA).

### Immunofluorescence microscopy

For vimentin and E-cadherin immunolocalization, cells were grown in Terasaki-type HLA wells (Nunc, Roskilde, Denmark), washed briefly in room temperature phosphate buffered saline (PBS) then fixed for 10 minutes in 4% paraformaldehyde (PFA). Cells were then washed for 3 × 10 min in PBS then blocked and permeabilized using 0.1% Triton X-100 made up in PBS containing 1% BSA and 0.2% sodium azide. Cells were then washed 3 times again for 10 min each in PBS, followed by the application of primary antibodies. For visualizing focal contacts by confocal microscopy, cells were grown on 35 mm diameter dishes (tissue culture grade, Nunc, Roskilde, Denmark), treated with antibody to paxillin as described above, then the base of the dish was cut out and inverted onto a drop of Vectorshield on a 24 × 50 mm coverslip and sealed with nail polish. Sources of all primary and secondary antibodies and their dilutions are detailed in Table 1. All antibodies were diluted in PBS with 1% BSA containing 0.2% sodium azide and incubated for at least 16 h at 4°C.

**Table 1:** Antibodies and probes used for fluorescent localization.

*Primary antibodies and probes*			
**Antibody/Probe**	**Species**	**Source**	**Dilution**

E-cadherin (HECD1)	Mouse, hybridoma supernatant	Dr. Alpha Yap (Inst. Mol. Sci., University of QLD)	1:2
Vimentin (V-9)	Mouse	Sigma-Aldrich	1:400
Paxillin (349)	Mouse	BD Biosciences	1:500
Phalloidin (F-actin)	Fungus	Molecular Probes (Invitrogen)	1:400

***Secondary antibodies***			

Anti-mouse: Alexa 488	Goat	Molecular Probes (Invitrogen)	1:2000

### Quantitative Real Time PCR (qRT-PCR)

All RNA extractions were performed using Trizol (Life Technologies, Gaithersburg, USA) according to manufacturer's instructions. This was followed by removal of contaminating genomic DNA using DNAse I (Promega Corporation, Sydney, Australia). The concentration and purity of RNA was determined using spectrophotometry (Nanodrop ND-1000 spectrophotometer, Thermo Scientific, Wilmington, USA) and depending on RNA yield, 0.1–1 μg of RNA was used for cDNA synthesis. cDNA synthesis was performed using superscript II reverse transcriptase (Invitrogen, Melbourne, Australia) according to manufacturer's instructions, using 10 nM dNTPs and the RNA degradation inhibitor RNAsin (Promega Corporation, Sydney, Australia). Quantitative determination of RNA levels of various genes was performed in triplicate using SYBR green (Applied Biosystems, Melbourne, Australia). The following pairs of primers (sense/antisense) were used: L32 (housekeeping gene), 5'-CAGGGTTCGTAGAAGATTCAAGGG-3'/5'-GATCGCTCACAATGTTTCCTCCAAG-3'; E-cadherin, 5'-GGCACAGATGGTGTGATTACAGTCAAAA-3'/5'-GTCCCAGGCGTAGACCAAGAAA-3'; Vimentin, 5'-GCTTCAGAGAGAGGAAGCCGAAAA-3'/5'-TTTCCAAGCCTGACCTCACGG-3'; MMP-2, 5'-CGGCCGCAGTGACGGAAA-3'/5'-CATCCTGGGACAGACGGAAG-3'; Zeb1/δEF1, 5'-GCCAATAAGCAAACGATTCTG-3'/5'-TTTGGCTGGATCACTTTCAAG-3'; Snail1, 5'-CTGCGGGAAGGCCTTCTCT-3'/5'-CGCCTGGCACTGGTACTTCTT-3'; Snail2, 5'-CGGACCCACACATTACCTTGTGTTT-3'/5'-CACAGCAGCCAGATTCCTCATGTTT-3'; Zeb2/SIP1, 5'-GGTCCAGATCGAAGCAGCTCAAT-3'/5'-GTGACTTCTATGTTTGTTCACATT-3'; RT-PCR and data collection were performed on an Applied Biosystems 5700 or 7300 analyser. All quantitations were normalized to expression of mRNA for the human ribosomal protein L32.

### Multiplex-tandem PCR (MT-PCR)

MT-PCR is a quantitative, 2-step process used to quantify expression levels of up to 72 genes in parallel. Selected cancer genes were pre-fabricated into Corbett Rotor discs from AusDiagnsotics Pty. Ltd. http://www.ausdiagnostics.com. The amplicon sequence for genes analysed using this method is provided [see Additional file 1]. MT-PCR was carried out according to the manufacturer's instructions. The first step performs a gene specific reverse transcriptase reaction followed by 15 cycles of amplification, after which insufficient product is formed to give significant competition between reactions in the subsequent individual PCR. The product from this reaction is then diluted 100 fold into individual PCR assays, each containing a single primer pair, using primers nested inside the amplicons of the first step. A Rotor-Gene (model 3000, Corbett) and Gene Disc Heat Sealer (Corbett) were used and additional reagents sourced from Quantace (MT-PCR analysis kit) through local supplier BioLine (NSW, Australia). Cycle threshold values (Ct) were determined for each reaction after visual inspection of melt curves for quality assurance using Corbett Rotor Gene 6000 series software Version 1.7 (Build 70).

### Statistical Analysis of Clinical Breast Cancer Dataset

All statistical analyses were performed using GraphPad Prism (GraphPad Software, California, USA).

## Results

### Staurosporine combined with EGF led to an enhanced EMT with a switch from Snail2 to Snail1 signalling

EGF plays an important role in the development of the normal breast and is also a key player in the progression of breast carcinomas, and as previously shown [27], EGF at 10 ng/ml over 72 h can induce EMT in PMC42-LA cells. In embryonic neural epithelial cells, ST rapidly reduced F-actin fibres and increased G-actin, delocalised cadherin, altered cell polarity, stimulated mesenchymal migration, and lead to expression of the HNK-1 marker of neural crest mesenchyme [32]. We sought here to test the actin-modifying agent ST alone and in combination with EGF in PMC42-LA cells.

**Figure 1:**
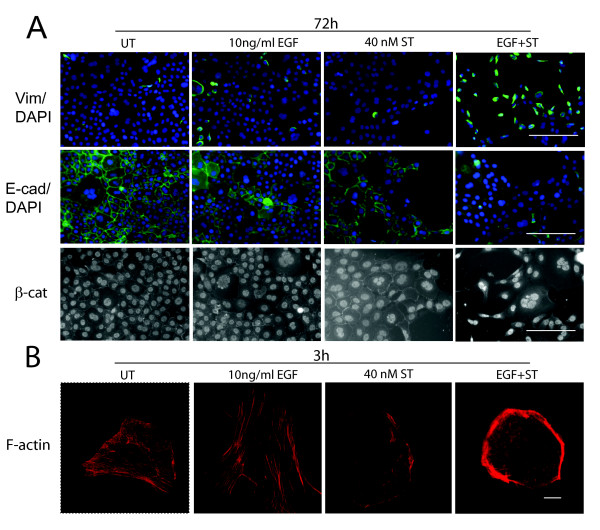
**ST augments and accelerates EGF-mediated EMT in PMC42-LA cells**. Immunostaining of cells treated with either 10 ng/ml EGF, 40 nM ST or both for 72 h, Vimentin: green, DAPI (nuclei): blue, E-cadherin: green, and β-catenin images taken at 10× magnification: scale bar = 200 μm. F-actin (red). Scale bar = 10 μM.

**Figure 2:**
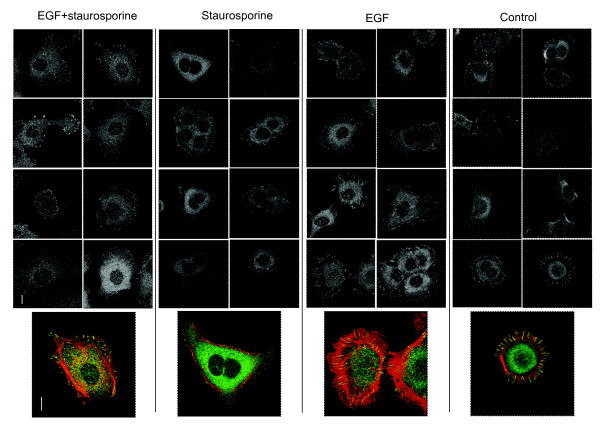
**ST rapidly reduces focal contact size in PMC42-LA cells**. Cells were treated for 3 h with 10 ng/ml EGF, 40 nM ST or both, fixed and immunostained for the focal contact specific protein paxillin (b+w, green in enlarged images) and F-actin (red). Magnification: ×63, scale bar = 10 μM.

**Figure 3:**
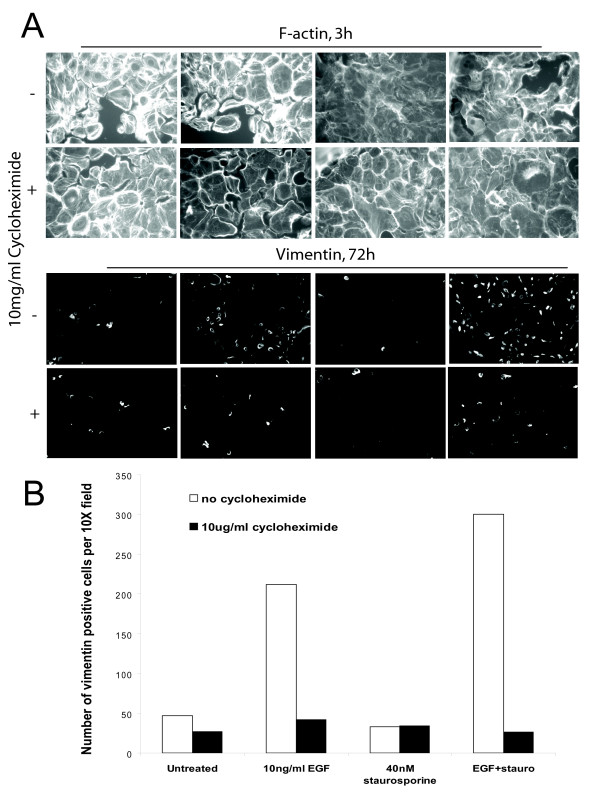
**Blockage of new protein synthesis did not block early actin changes**. 10 mg/ml cycloheximide did not affect early (3 h) loss of actin stress fibres (A) but significantly reduced vimentin induction by EGF and EGF+ST (72 h) (A, B).

As shown in Figure 1, 40 nM ST combined with 10 ng/ml EGF over 3 days led to an enhanced EMT, with a more marked increase of vimentin protein and reduction of E-cadherin expression by immunostaining than with EGF alone. Non-phosphorylated β-catenin stained brightly in the nucleus in the combined treatment rather than at the cell membrane, indicating activation of the Wnt pathway, consistent with an EMT. Rapid (3 h) F-actin disruption and depolymerisation was due to the ST component, as this was not found in cells treated with EGF alone, but was present in ST alone and in the combined treatment (Figure 1). Focal contact size was reduced by ST. This was shown by immunostaining for the focal contact protein paxillin (Figure 2, individual black and white images: paxillin, colour images: F-actin – red, combined with paxillin in green). FAK immunostaining at focal contacts was also visibly reduced by ST [Additional file 2]. The early actin changes were not affected by the protein synthesis inhibitor cycloheximide, consistent with post-translational mechanisms (Figure 3). In contrast, vimentin induction was significantly reduced by cycloheximide, suggesting *de novo *production consistent with an induced EMT program.

**Figure 4:**
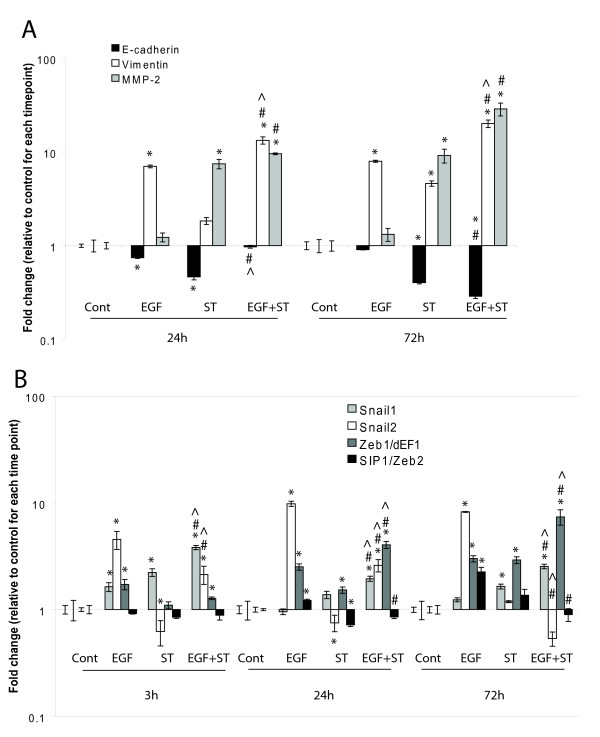
**QRT-PCR of PMC42-LA cells treated for 72 h with 10 ng/ml EGF, 40 nM ST or both**. Control: untreated (A) E-cadherin mRNA expression plotted against increased expression of hallmark mesenchymal genes MMP-2 and vimentin. (B) Comparison of expression of four E-cadherin repressor genes in the treatment regime used in A. Error bar values are standard error. Significance was set at p </= 0.05. * symbol indicates a statistically significant difference from the control within each time point. In the combined treatment, ^ and # indicate statistical significance from ST and EGF individual treatments, respectively, within each time point.

QRT-PCR data (Figure 4) confirmed the immunohistochemical results shown in Figure 1: Vimentin and MMP-2 mRNA were upregulated, and E-cadherin mRNA downregulated, both to a greater degree by the combined treatment. EGF alone induced vimentin, whereas ST induced MMP-2 and repressed E-cadherin more strongly than EGF alone (Figure 4A). These changes were largely apparent by 24 hours, and remained or increased by 72 hours. The effect of combined EGF and ST on reducing E-cadherin expression was not seen at 24 hours, but was prominent at 72 hours.

Snail1 and Snail2, amongst other E-cadherin repressors such as Zeb1/δEF1 and Zeb2/SIP1, are transcription factors which orchestrate EMT because their induction leads to a cascade of downstream gene expression changes which embody and reinforce the EMT phenotype. As shown in Figure 4B, EGF almost exclusively induced Snail2 at the early stage examined (3 h), whereas ST induced Snail1 at this time point. When EGF and ST were combined, at 3 h, the relative expression pattern for Snail family genes was dominated by ST. Some Snail2 was still expressed along with Snail1 at 3 h and 24 h, however by 72 h the expression of Snail1 predominated. Snail2 persisted as the main Snail gene expressed in EGF-treated cultures, and Snail1 in the ST-treated cultures. The Snail1 downstream effector gene, Zeb1/δEF1 [10] followed Snail1 induction, being maximally induced at 72 h in all treatments, but the expression level was greatest in those receiving combined treatment. Increased Zeb1/δEF1 was also seen following Snail2 induction in EGF-treated cells. Although Zeb2/SIP1 was somewhat induced by EGF at 24 h and at 72 h, and also by ST at 72 h, it was repressed by EGF+ST at all treatment times.

### Staurosporine and EGF individually induce diverse gene expression profiles

**Figure 5:**
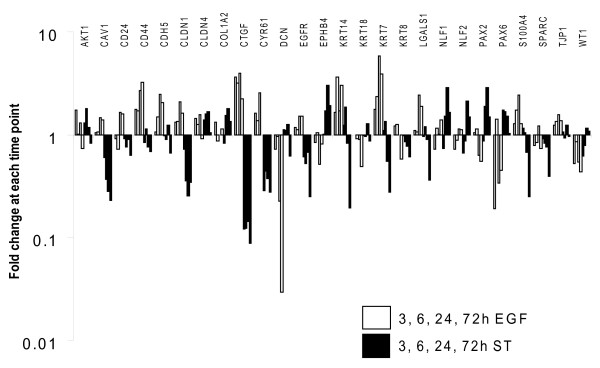
**MT-PCR of PMC42-LA cells treated for 3, 6, 24 or 72 h with ST or EGF**. Expression data for a range of various genes with relevance to EMT are shown as fold change i.e. corrected for untreated cells at each time point for each gene.

The gene expression programmes induced by ST and by EGF alone were dramatically different over the 72 h period in PMC42-LA cells when examined by MT-PCR (Figure 5). Genes induced early (3–6 h) by EGF alone were AKT1, CYR61, CLDN4, CD44, CLDN1, CTGF, KRT14, KRT7 and S100A4. Expression of the last 6 genes in this list persisted at later timepoints (24–72 h) whereas genes primarily induced late were CAV1, CD24, CDH5, EGFR and LGALS1. Genes which were repressed early by EGF included CD24, DCN, EPHB4, KRT18, NLF1, NLF2, SPARC and WT1, with DCN, EPHB4, KRT18, SPARC and WT1 repression persisting at later timepoints. Genes mainly repressed only in the later time period (24–72 h) by EGF alone were KRT8, PAX2 and PAX6. Genes induced early by ST differed to those induced by EGF at this timepoint. They included PAX6, DCN and EPHB4, where the latter two genes were repressed at the early timepoints by EGF. Genes mainly induced late by ST but repressed by EGF at this timepoint included EPHB4, PAX2, PAX6 and WT1. In addition, ST repressed genes early which were induced by EGF: CLDN1, CTGF, CYR61 and more pertinent to the EGF signal transduction cascade, EGFR. ST repressed this gene in the "late" grouping, along with CTGF, CYR61 and CAV1. Overall gene changes mediated by ST or EGF alone are summarized in Table 2.

**Table 2:** Summary of gene expression changes determined by MT-PCR.

	Treatment	
**Gene name**	**EGF**	**ST**
CAV1	+	-
CD24	+	NC
EGFR	+	-
EPHB4	NC	+
NLF1	NC	+
NLF2	NC	+
CD44	+	-
CDH5	+	-
CLDN1	+	-
CTGF	++	--
CYR61	+	-
KRT7	+	-
LGALS1	+	-
S100A4	+	-

"+" or "-" denotes whether genes were predominantly induced or repressed over 72 h treatment. "NC" – no change.

### Clinical implication of Snail1 expression in human primary breast tumours

**Figure 6:**
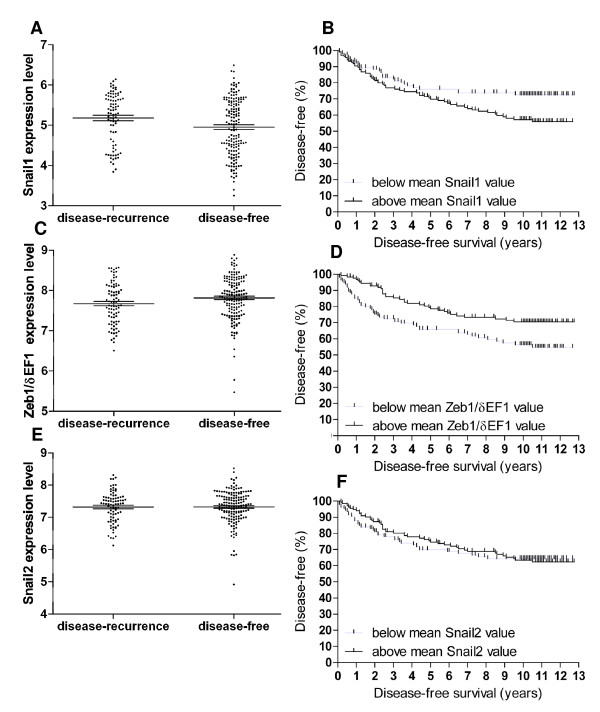
**Statistical interrogation of the GSE4922 microarray dataset of human primary breast cancers**. Expression levels of Snail1 (A), Zeb1/δEF1 (C) and Snail2 (E) were compared between tumours from patients who remained disease-free and those from patients who had a disease-recurrence event (local, regional, distant or death from breast cancer) across the 242 patients with a non-zero disease-free survival time in the GSE4922 microarray dataset [39]. Snail1 was expressed more highly in tumours from patients who had a disease-recurrence event, as determined by Mann-Whitney test (p = 0.017). Zeb1/δEF1 was expressed more highly in tumours from patients who remained disease-free, as determined by Mann-Whitney test (p = 0.026). Kaplan-Meier analysis of disease-free survival comparing patients with a Snail1 (B), Zeb1/δEF1 (D) or Snail2 (F) expression level above or below the mean expression level. Overall patients with an above mean Snail1 expression value had a poorer clinical outcome as determined by Gehan-Breslow-Wilcoxon test (p = 0.031), as did those patients with a below mean Zeb1/δEF1 expression value as determined by Gehan-Breslow-Wilcoxon test (p = 0.004).

This study using the PMC42-LA human breast carcinoma cell line indicated that under the conditions studied, the most intense EMT was related to a heightened expression of Snail1 rather than Snail2, although both led to increased Zeb1/δEF1 with highest Zeb1/δEF1 achieved with a combination of both treatments. Accordingly, we interrogated the publicly available GSE4922 dataset of microarrays performed on primary breast cancer from a large cohort of breast cancer patients with long-term follow-up [39], for evidence of clinical implications due to increased expression levels of these E-cadherin repressors. As shown in Figure 6A, across the 242 patients with non-zero disease-free survival times, we found Snail1 differentially expressed in tumours from patients that remained disease-free compared with those from patients in which there was either a local, regional or distant disease recurrence event, or death from breast cancer (Mann-Whitney test, p = 0.017). A Kaplan-Meier analysis comparing patients with Snail1 expression values either above or below the mean Snail1 expression value (Figure 6B) revealed that patients with tumours exhibiting higher Snail1 expression had a higher rate of disease recurrence (two-tailed Gehan-Breslow-Wilcoxon test, p = 0.031). As shown in Figure 6C, one probeset for Zeb1/δEF1 demonstrated differential expression in regards to disease recurrence/death from breast cancer (Mann-Whitney test, p = 0.026). A Kaplan-Meier analysis comparing patients with Zeb1/δEF1 expression values either above or below the mean Zeb1/δEF1 expression value (Figure 6D) revealed that patients with tumours exhibiting lower Zeb1/δEF1 expression had a higher rate of disease recurrence (two-tailed Gehan-Breslow-Wilcoxon test, p = 0.004). No significant differences were seen in relation to Snail2 expression and outcome (Figure 6E and 6F).

## Discussion

In this study we have shown that early Snail1 rather than Snail2 expression led to the most pronounced EMT response in the breast carcinoma cell line PMC42-LA. Providing further support for a contributing role for Snail1 in invasive breast cancer, we have presented analysis from a large cohort of breast cancer patients associating increased Snail1 mRNA expression with increased risk of disease recurrence. The analysis presented here, associating higher tumoural Snail1 expression with increased risk of disease recurrence, adds to the growing body of evidence implicating Snail1 in human breast cancer progression [40-45]. While our analysis was restricted to overall levels of Snail1 mRNA, Snail1 protein has been selectively localised to the tumour-stroma interface [46]. Snail2 did not correlate with an increased risk of recurring disease in our analysis, however other studies have linked Snail2 as a parameter of disease aggressiveness in ovarian and breast cancer, and with poor survival in colorectal cancer [43,47]. Zeb1/δEF1 is a predictor of poor prognosis of uterine cancer and is expressed in colorectal cancer [48,49], however a statistical correlation of Zeb1/δEF1 with breast cancer progression has not been reported, despite an association of its' histological expression with increasing breast tumour grade [50]. We found an inverse correlation between Zeb1/δEF1 expression and outcome, with lower levels associating with a worse outcome. This was surprising because we found a longer-term Zeb1/δEF1 response to both the ST/Snail1 and EGF/Snail2 EMT pathways. One possibility is that Zeb1/δEF1 expression may be more strongly influenced by stromal expression, as reported by Aigner *et al *[50]. While these disparate data do not preclude a role for all three E-cadherin repressor genes in breast cancer progression, perhaps in our analysis in a subset of cells masked by the general population, Snail1 appears outstanding among the E-cadherin repressors in terms of its clinical relevance.

To some extent this preferential emphasis on Snail1 is surprising. Snail1 and Snail2 display overlapping roles in EMT in normal embryonic and breast development and are upregulated to varying degrees in invasive carcinogenesis [41,43,51]. In human breast cancer biopsies a strong correlation was found between Snail1 expression, reduced E-cadherin expression and invasive grade of tumours [41-43]. Snail1 expression has been found in infiltrating ductal carcinomas associated with lymph node metastases [41,52] and distant metastases including effusions [43,45], and has been associated with recurrence of breast carcinomas [44]. Snail2 expression is also associated with the presence of tumour effusions, metastasis and recurrence [43,53], but is also associated with partially differentiated breast cancers [45], perhaps reflective of the role of Snail2 in the developing breast [42]. Their ability to initiate EMT depends upon their ability to repress E-cadherin transcription and, at least for Snail1, sustain this effect despite continual signals targeting it for degradation at the proteasome [54]. The preferential upregulation of Snail1 in the ST and ST+EGF treated cells may reflect a specific set of triggers which drive EMT expression in breast cancers, in comparison to those induced with EGF alone.

### Snail1 induces Zeb1/δEF1

Snail1 induction with ST and with EGF+ST declined by 72 h, however it was replaced by Zeb1/δEF1 expression, which was induced increasingly to the longest time point, when E-cadherin was also maximally repressed. It appears that Zeb1/δEF1 maintains long term repression of E-cadherin initiated by Snail1, as suggested by others previously [10,15]. Snail1 is sensitive to GSK-3β phosphorylation targeting it for degradation, therefore it is highly unstable with a half-life of approximately 25 minutes [54]. However Snail1 induces Zeb1/δEF1 in various epithelial cell lines, and only in the cell lines where Snail1 and Zeb1/δEF1 were co-expressed was E-cadherin expression strongly downregulated [10]. In that study, Zeb1/δEF1was activated by Snail1 after a lag period, and Zeb1/δEF1 activation was maintained even after Snail1 expression declined. This has been shown functionally in the progression of breast carcinomas, where Snail1 is necessary for initial invasion, and Zeb1/δEF1 and other E-cadherin repressor genes are involved in the maintenance of the mesenchymal phenotype, including motility [15]. Our analysis of Affymetrix expression data from 51 human breast cancer cell lines also show Zeb1/δEF1 to correlate most tightly with the mesenchymal/BasalB subgroup [55]. Coupling of Snail1 and Zeb1/δEF1 induction was also found when CCN6 (Wnt-1-induced signalling protein 3) was knocked down in mammary epithelial cells leading to E-cadherin downregulation [56]. Such progression from Snail1 to Zeb1/δEF1 has not been reported for Snail2 [15]. In keeping with Zeb1/δEF1 induced by Snail1 after a lag period, the conditions leading to early (3 h) expression of Snail1 in preference to Snail2 in the current study led to the strongest expression of Zeb1/δEF1, which peaked at 72 h, the time point at which E-cadherin was most repressed (Figure 3A). Interestingly, in the mesenchymal counterpart PMC42-ET cell line, in which the expression of E-cadherin and several other epithelial genes are strongly repressed, Zeb1/δEF1 mRNA expression is ~13-fold higher than in the epithelial PMC42-LA cells (data not shown). Combined, the results in the current study indicate that Snail1 could mediate E-cadherin repression in EGF+ST induced EMT in PMC42-LA cells through Zeb1/δEF1 expression. The early increase in Snail2 by EGF was followed by upregulation of Zeb1/δEF1, however, this was to a lesser extent than either ST alone or ST+EGF. It remains to be seen whether the regulation of Zeb1/δEF1 involves the miR-200 family and miR-205, recently shown to regulate TGFβ-induced EMT of mammary and ovarian epithelial cells by targeting Zeb1/δEF1 and SIP1, and shown to be downregulated in regions of breast cancer specimens with E-cadherin loss. Although somewhat upregulated by individual treatments with EGF or ST, Zeb2/SIP1 was repressed in the combined treatment at all timepoints (fig 4) suggesting this Zeb family member is less likely to be involved in Snail1-driven breast cancer EMT.

### Targets of staurosporine

ST is a potent, cell-permeable broad-spectrum inhibitor of protein kinases, inhibiting CaM kinase, MLCK (myosin light chain kinase), PKA, PKG and PKC, the latter being the most sensitive target (Calbiochem Inhibitor Sourcebook). ST at high doses (>50 nM) induces apoptosis [57-59] and at low doses (<50 nM) can lead to differentiation, such as in granulosa tumour cells [60]. In the current study, the concentration of ST used did not significantly induce apoptosis (data not shown).

### Staurosporine and EMT

We have proposed that ST-induced EMT of neural epithelia was initiated rapidly due chiefly to inhibition of atypical PKC (aPKC) [61]. In other cell systems, aPKC is a crucial component of the nexus between F-actin bundles, Par-3/6, Cdc42 and the tight junctional complex, the latter collaborating to maintain the integrity of adjacent cadherin junctions. ST induced Snail1 early (3 h) in PMC42-LA cells, coinciding with F-actin depolymerization and the reduction of focal contact size. These cytoskeletal and adhesive events did not require new protein synthesis, this being consistent with functional protein modifications due to alteration in phosphorylation. A change in focal contact size through the alteration of actin may stimulate rapid changes in integrin signaling. Integrin linked kinase (ILK) is a component of focal adhesion plaques [62,63], and has direct effects on actin modification through the phosphorylation of cofilin, where it acts to block the actin-severing effect of unphosphorylated cofilin [64], and integrin stimulation triggers signaling through this kinase [65,66]. In breast cancer cells, ILK upregulation confers resistance to anoikis [67], increases tumour invasion, and rapidly induces Snail1 [68-70]. Given the specific role ILK has in inducing the transcription of Snail1 it is possible that ST induces Snail1 through this pathway.

### Staurosporine mediated effects on ECM cellular attachment

A reduction in focal contact size mediated by ST may have achieved cell-ECM loosening which is a prerequisite for EMT [71] and hence ST when combined with EGF may have accelerated the EMT program through this mechanism. Another pathway of actin reorganization may also have been triggered by early transcription-independent mechanisms. EphB4 was specifically upregulated after 6 h by ST (and repressed by EGF), a gene which re-organizes the actin cytoskeleton to promote cell movement [72]. Additionally, NLF1 and 2, factors which have been shown to induce genes which control cellular architecture and ECM adherence (Rnd1 and GEM GTPase) [73], were specifically and dramatically upregulated by ST compared to EGF (Figure 5). Promoters of the NLF genes contain NFkB binding sites and they were found to mediate NFkB effects in endothelial cells treated with proinflammatory cytokines IL1B and TNFα [73].

### NFkB and Zeb1/δEF1

NFkB induces Zeb1/δEF1 in EMT. Constitutive expression of the NFkB subunit p65 specifically induced Zeb1/δEF1 and Zeb2 in the mammary cell line MCF10A, concomitant with suppression E-cadherin and induction of an EMT, and transient transfection of p65 increased transcription of the Zeb1/δEF1 promoter [74]. The induction of NLF genes mainly by ST in the current study implies the activation of NFkB, which may have contributed to Zeb1/δEF1 induction at 72 h in ST and ST+EGF treated PMC42-LA (Figure 4). This pathway of NFkB- Zeb1/δEF1 induction may act to further enhance the initiated ILK-Snail1 induction pathway (described earlier), with potential cross-induction of NFkB by ILK [75] culminating in the late Zeb1/δEF1 induction observed in figure 4 in the combined treatment.

### Effect of EGF on gene expression (MT-PCR)

EGF alone promoted a specific gene profile when examined by MT-PCR (Figure 5), including an increase in CAV1. Stimulation of epithelial cells with EGF has been shown to result in a profound increase in the number of caveolar structures at the plasma membrane, along with Src-phosphorylation of CAV1 on tyrosine 14 [76], and indeed EGF induced CAV1 mRNA in a study examining the effect of the PPARgamma ligand rosiglitazone on phosphorylation of the EGFR [77]. In the current study, CTGF, its relative CRY61, (related through conserved cysteine rich domains both with distinct roles in modulating ECM signalling) and the calcium binding protein S100A4 were upregulated by EGF and not by ST. CTGF is elevated in advanced stages of breast cancer, promotes mesenchymal features in MCF-7 cells when overexpressed, and is an upstream inducer of S100A4, a gene important for its positive effects on cell migration [78]. The CTGF family member, CYR61, was also specifically upregulated by EGF, as has been demonstrated in MCF-7 cells treated with EGF [79,80]. Interestingly, the ST-repressive effect on CTGF and CYR61 expression observed in the current study was also seen in an analysis of gene expression during ST-induced neuronal differentiation of human prostate cancer cells [81] in which ST was shown to have a role in neuronal differentiation and inhibition of malignancy. Only a small number of genes were regulated by EGF or ST in the same manner. These were CLDN4 (up), SPARC and WT1 (both down). While more needs to be learnt about the roles of these downstream effectors, the dramatically different and often opposite profiles seen in the current study clearly illustrate that EGF and ST are operating in two very different ways.

## Conclusion

These studies highlight the importance of Snail1 in EMT in the breast carcinoma cell line PMC42 and in the progression of breast carcinoma *in vivo*. The breast ductal epithelium is a complex environment therefore it is unlikely that EGF initiates or acts alone in regulating breast cancer cell invasion. EGF may combine with other physiological factors which alter the actin cytoskeleton, as achieved here using ST, producing a similar cross-modulation and rapid EMT. Indeed, epigenetic signaling from the microenvironment leading to changes in cellular cytoskeletal architecture has been implicated in drawing tumour cells from dormancy to metastatic growth [82]. Further study is needed to ascertain how this system is at work in invading primary breast tumours.

## Abbreviations

EMT: epithelial to mesenchymal transition; ST: staurosporine; aPKC: atypical protein kinase C; EGF: epidermal growth factor; MT-PCR: multiplex tandem PCR; ECM: extracellular matrix; EGFR: epidermal growth factor receptor; CAV1: caveolin 1; CTGF: connective tissue growth factor; CYR61: cysteine-rich, angiogenic inducer, 61; CD44: CD44 antigen (Indian blood group); S100A4: S100 calcium binding protein A4; SNAI2: Snail2; NLF: nuclear localized factor; EPHB4: EPH receptor B4; FGF: fibroblast growth factor; Wnt: wingless-type; BMP: bone morphogenic protein; HGF: hepatocyte growth factor; IGF: insulin growth factor; TNFα: tumor necrosis factor alpha; PI3K: phosphoinositide 3 kinase; TGFβ: transforming growth factor beta; MAPK: mitogen activated protein kinase; AKT: v-akt murine thymoma viral oncogene homolog 1; CLDN: claudin; CD24: CD24 molecule; CDH: cadherin; LGALS1: lectin, galactoside-binding, soluble, 1; DCN: decorin; SPARC: secreted protein, acidic, cysteine-rich (osteonectin); WT1: Wilms tumor 1; PAX: paired box gene; KRT: keratin; MLCK: myosin light chain kinase; PKA: protein kinase A; PKG: protein kinase G; ILK: integrin linked kinase; NFkB: nuclear factor-kappa B.

## Competing interests

The authors declare that they have no competing interests.

## Authors' contributions

HH carried out immunohistochemistry, confocal microscopy and quantitative real time PCR, MT-PCR timepoint analyses for EGF alone, and drafted the manuscript, RW performed MT-PCR for ST alone, TB performed MT-PCR calculations and statistical analyses of the breast tumor patient database, EWT and DFN co-participated in the design and co-ordination of the study originally conceived by DFN. EWT designed the MT-PCR assays and introduced the analysis of clinical samples. All authors read and approved the final manuscript.

## Pre-publication history

The pre-publication history for this paper can be accessed here:

http://www.biomedcentral.com/1471-2407/9/235/prepub

## Supplementary Material

Additional file 1 **Amplicon sequences for genes analysed using MT-PCR**. The sequences provided represent the individual gene fragments amplified by MT-PCR. Click here for file

Additional file 2 **ST rapidly reduces focal contact size in PMC42-LA cells**. Untreated cells and cells treated for 3 h with 40 nM ST were fixed and immunostained for the focal contact specific protein Focal Adhesion Kinase (FAK) (b+w, green in merged images) and F-actin (red). Scale bar = 10 μM. Click here for file
