# Supplementary figures and images for: Staurosporine augments EGF-mediated EMT in PMC42-LA cells through actin depolymerisation, focal contact size reduction and Snail1 induction – A model for cross-modulation
Source: BMC Cancer. 2009 Jul 15;9:235. doi: 10.1186/1471-2407-9-235 (PMC2717979; doi:10.1186/1471-2407-9-235)

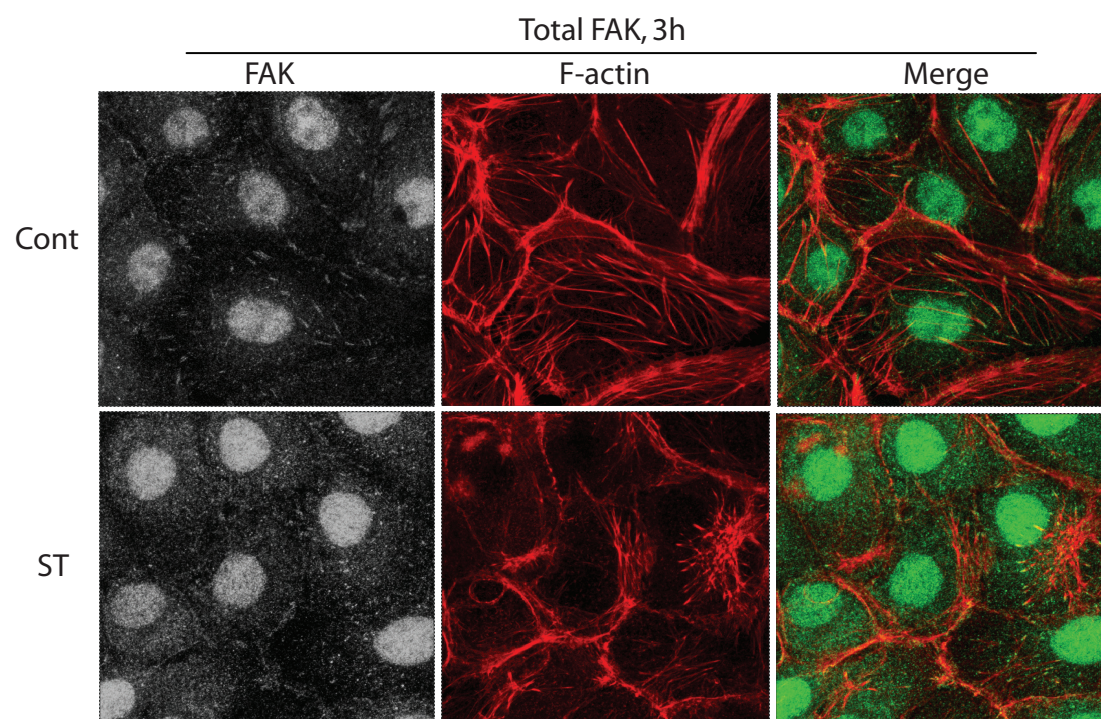

Supplement: Additional file 2 — ST rapidly reduces focal contact size in PMC42-LA cells. Untreated cells and cells treated for 3 h with 40 nM ST were fixed and immunostained for the focal contact specific protein Focal Adhesion Kinase (FAK) (b+w, green in merged images) and F-actin (red). Scale bar = 10 μM. [file 1471-2407-9-235-S2.pdf]
